# Surgery on aggressive fibroma of the posterior compartment of the knee: A case report

**DOI:** 10.1016/j.ijscr.2019.10.060

**Published:** 2019-11-06

**Authors:** R.M.S.N. Magetsari, M.N.S. Irawan

**Affiliations:** Department of Orthopaedics and Traumatology, Faculty of Medicine, Padjadjaran University, Bandung, Indonesia

**Keywords:** Aggresive fibroma, Popliteal, Resection

## Abstract

**Introduction:**

Fibroma is a benign tumor. More than 99% fibroma arises from tendon sheaths or tendons. Aggressive Fibroma at posterior compartment knee is a rare case. This report presents one case of fibroma at posterior compartment of the knee with the treatment.

**Case report:**

A boy with 4-year history of knee discomfort and lump at posterior knee joint, no history of infection and trauma. Physical examination revealed a mass (10 × 9 × 7 cm) with limited Range of Motion extension 30°, flexion 70°. MRI revealed a soft tissue mass at posterior knee compartment. Histologic examination showed a circumscribed and lobulated hypo cellular mass containing spindle cells diagnosed as aggressive fibroma. The patient underwent popliteal resection. An ellipse incision was made 2 cm margin of skin around the biopsy site. Fasciocutaneous flaps were created and retracted to expose the posterior compartment. The tumor had a partial well-defined capsule, popliteal artery and vein embedded and pressing the sciatic nerve. The tumor and enveloping muscles were elevated from the base of the compartment. The sciatic nerve was preserved with epineurotomy. The popliteal artery was reconstructed with saphenous veins graft. After the tumor was resected, the heads of gastrocnemius were sutured to each other and to the hamstring muscles to cover popliteal space. After one year postoperative, there was a good vascularization, no recurrence and neurological deficit with Musculoskeletal Tumour Society Scoring System 80%.

**Conclusion:**

One year postoperative with popliteal resection surgery yields a good result. A follow-up is needed for risk of recurrence and malignancy.

## Highlights

• We report a rare case of aggressive fibroma at posterior compartment of the knee.
• The tumor was resected from the posterior compartment with the preservation of neurovascular structures around the tumor.
• The popliteal space is covered with the heads of gastrocnemius sutured to the hamstring muscles.
• One year postoperative, the vascularization was good, no recurrence and neurological deficit with MSTS 80%.

## 1 Introduction

Fibroma is a rare and benign soft-tissue tumor that usually originates from tendon or tendon sheaths in fingers, toes, and wrist joints. About 99% of fibromas arise from the tendon sheaths or tendons, and rarely occur in the joint capsules [[1], [2], [3]]. This fibroma is characterized histologically by a dense fibrocollagenous stroma with scattered spindle-shaped fibroblasts and narrow slit-like vascular spaces [1,4]. There was only a limited number of reports that have been published on the uncommon and unusual lesions, especially in the knee. Fibroma of the knee has very rarely been reported before, only 26 cases reported worldwide [1,[5], [6], [7], [8], [9], [10], [11], [12], [13], [14], [15], [16], [17], [18], [19]]. We report a case of fibroma at posterior compartment of the knee. This article has been reported in line with the SCARE criteria, and written informed consent was obtained from the patient’s parents for publication of this case report and accompanying images [20].

## 2 Case report

**Fig. 1:**
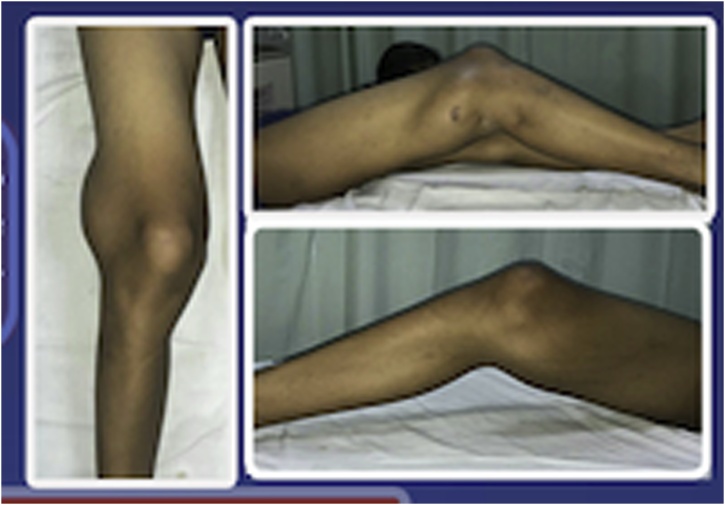
Lump at the posterior knee joint with limited range of motion.

A 14-year-old boy presented to our orthopaedic oncology clinic with 4-years history of knee discomfort and lump at posterior knee joint. There was no history of infection and trauma before. A physical examination revealed a mass (sized 10 × 9 × 7 cm) with limited range of motion (ROM) extension 30°, flexion 70° (Fig. 1).

**Fig. 2:**
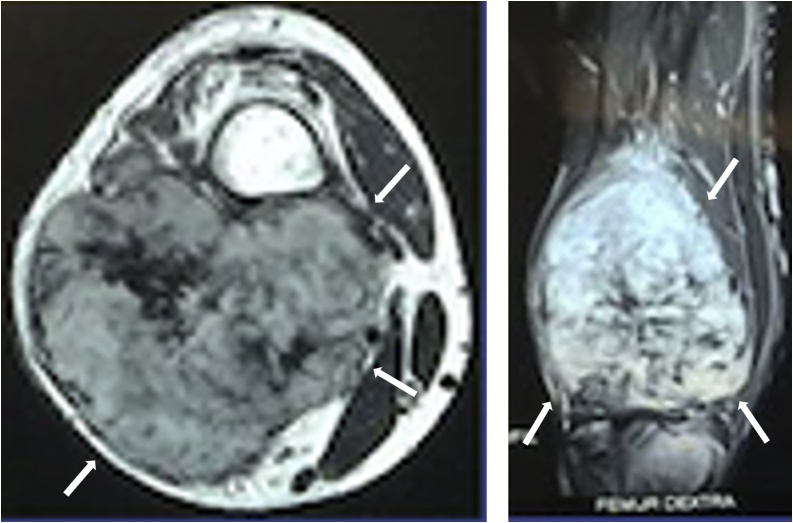
MRI revealed soft tissue mass sized 10 × 9 × 7 cm at posterior compartment of knee (white arrow).

**Fig. 3:**
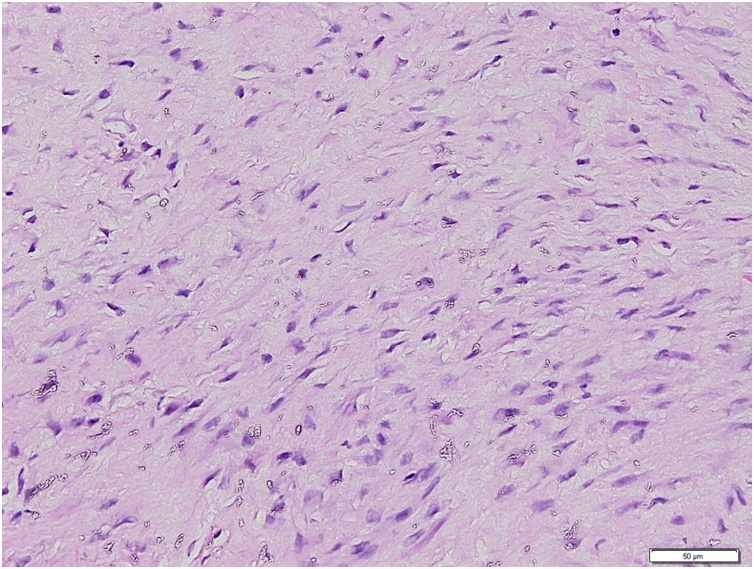
Histological examination showed a circumscribed and lobulated hypo cellular mass containing spindle cells.

Magnetic resonance imaging (MRI) revealed a soft tissue mass at the posterior compartment of the knee (Fig. 2). Histologic study was taken from core biopsy examination and the result showed a circumscribed and lobulated hypo cellular mass containing spindle cells (Fig. 3). The diagnosis was aggressive fibroma.

**Fig. 4:**
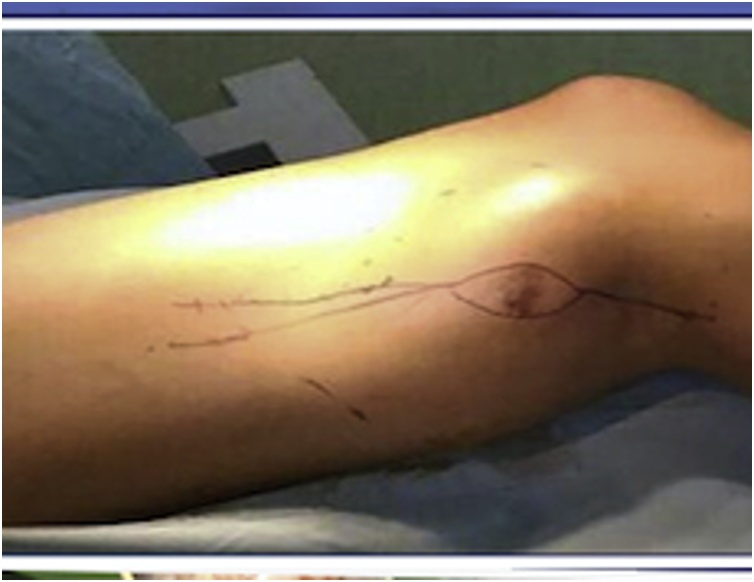
The ellips incision was made around the biopsy site.

**Fig. 5:**
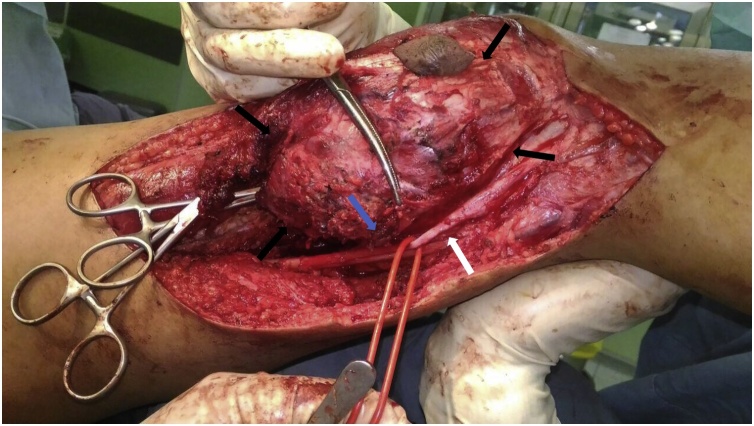
Tumor mass was embbeded in the posterior compartment of the knee.

**Fig. 6:**
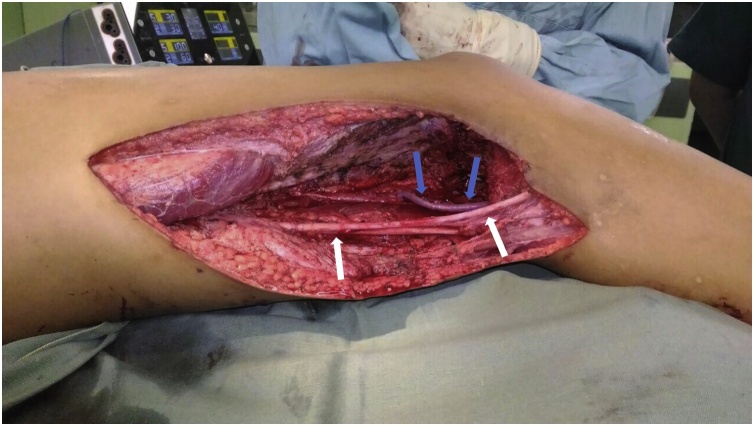
The popliteal artery was reconstructed with saphenous vein graft.

The patient underwent popliteal resection. The ellipse incision was made 2 cm margin of skin around the biopsy site (Fig. 4). Fasciocutaneous flaps were created and retracted to expose the posterior compartment. Tumor mass had a partial well-defined capsule, popliteal artery and vein was embedded, and pressing the sciatic nerve (Fig. 5). The tumor mass and enveloping muscles were elevated from the base of the compartment and the sciatic nerve was preserved with epineurotomy. The popliteal artery was reconstructed with saphenous veins graft (Fig. 6). After tumor resected, the heads of gastrocnemius were sutured each other’s and to the hamstring muscles to covered popliteal space. After one year postoperative, vascularization was good, no recurrence and neurological deficit with Musculoskeletal Tumour Society Scoring System (MSTS) 80% [21].

## 3 Discussion

Fibroma is a rare and benign soft tissue tumor that usually arises in tendons or tendon sheaths [5]. It typically develops in young, adult males with peak of incidence in the third to fourth decade [1]. It is commonly present as a painless, slow growing solid nodule [1,6,7]. The most common lesions involve the fingers, hands, or wrists and the less common locations are in the toe, foot, ankle, leg, knee, forearm, elbow, temporomandibular joint, chest, and back [1,7,8,22,23]. In the knee region, localized nodular synovitis, pigmented villonodular synovitis, and synovial chondromatosis can cause swelling, pain, and limitation of knee ROM. Other rare causes including hemangioma, lipoma, and tenosynovial fibroma [24]. It is unclear whether fibromas are the result of reactive proliferation or true neoplasms [17]. However, it is accepted that this tumor is a slowly growing fibrous nodule that is attached to the tendon sheath. Fibromas in the knee region is very rare with less than 30 cases being reported worldwide. Our case was located in the posterior compartment of the knee joint with involvement of popliteal artery and vein, and the surrounding muscles.

Imaging studies with MRI have been reported before. Generally, the T1-weighted MRI typically show a well-defined lesion with homogenous low or iso-signal intensity compared with the muscle. On the T2-weighted images, some cases show a mixture of low and high signal intensity. In our case, the MRI showed a large mass with the size of 10 × 9 × 7 cm in the posterior compartment of the knee.

Regarding the histology findings, fibromas infrequently contain hemosiderin-laden macrophages, xanthoma cells or multinucleated giant cells, which in contrast to Giant Cell Tumor of Tendon Sheaths and Pigmented Villonodular Synovitis [25,26]. Nodular fasciitis is also rare in the knee joint and has a similar histology to a fibroma, but usually consists of a rapidly growing mass [17,27,28]. In our case, we found a circumscribed and lobulated hypo cellular mass containing mainly a spindle-shaped cells which suited to the histology of fibroma.

Regarding the treatment of fibromas of tendon sheath, the lack of cases made it difficult to build a consensus. The prognosis after marginal excision of these lesions is generally good due to their slow growth and benign histologic appearance. Chung et al. found a local recurrence rate of 24% after excision [1]. However, Moretti et al. stated in their study that none of the knee cases they studied before have reported any recurrence of the tumor [5]. These statements are corresponded to our case, one year after the tumor was resected the patient showed a good vascularization, neither sign of recurrence nor neurological deficit. Our study also showed a good functional outcome with Musculoskeletal Tumour Society Scoring System (MSTS) 80% [21].

## 4 Conclusion

In summary, fibroma of the knee is a very rare benign soft-tissue tumor. It typically presents as a painless, slow growing, solid nodule. Fibroma should be included in a differential diagnosis of a soft tissue tumor arising from the knee joint. However, the tumor must be excised completely. In our case, one year postoperatively the patient showed a good result but a careful follow-up is needed to observe the risk of recurrence and malignancy.

## Declaration of Competing Interest

Both authors have nothing to disclosed.

## Sources of funding

Both authors have no source of funding for this article.

## Ethical approval

Ethics approval and consent to publish has been obtained from our institution.

Reference number: LB.04.01/A05/EC/035/II/2018.

## Consent

Written informed consent was obtained from the patient’s parents for publication of this case report and accompanying images. A copy of the written consent is available for review by the Editor-in-Chief of this journal on request.

## Author contribution

All authors performed the surgery.

All authors designed and drafted the manuscript.

All authors have read and approved the final version of the manuscript

## Registration of research studies

This case report is not a “first-in-man” study, thus we did not register the research study.

## Guarantor

1. Magetsari M.R.N., MD

2. Irawan M.N.S, MD

Both authors accept full responsibility for the work and/or the conduct of study.

## Provenance and peer review

Not commissioned, externally peer-reviewed
